# Necrobiotic granulomas of the bowel accompanying 3-year postsurgical recurrence of colon cancer: A case report

**DOI:** 10.1016/j.ijscr.2020.02.014

**Published:** 2020-02-07

**Authors:** Ali Al Khader, Esra Nsour, Raed Aldabbas, Anwar Alneweiri

**Affiliations:** a Faculty of Medicine, Al-Balqa Applied University, P.O. Box 19117, Al-Salt, Jordan; b Department of Pathology, Al Hussein Salt Hospital, Ministry of Health, Al-Salt, Jordan; c Private Sector, Jordan; d Al Hussein Salt Hospital, Ministry of Health, Al-Salt, Jordan

**Keywords:** Cancer, Colon, Granuloma

## Abstract

**Introduction:**

Postsurgical necrobiotic granulomas present a diagnostic challenge and have been described in organs such as the urinary bladder, prostate, and uterus. However, occurrence of these granulomas is rare in the bowel.

**Presentation of the case:**

In this report, we present the case of a 28-year-old woman with recurrent multifocal colonic adenocarcinoma 3 years after sigmoidectomy. Intraoperatively, the ileum was suspicious for tumor deposits. Total colectomy with segmental small bowel resection was performed, and the histopathological findings of the small bowel specimen were consistent with necrobiotic granulomas with no evidence of malignancy. Infectious disease workup was negative. The patient was administered adjuvant chemotherapy and had no negative outcomes at 8 months after the surgery.

**Discussion:**

This is a rare and challenging case of postsurgical necrobiotic granulomas of the bowel.

**Conclusion:**

Granulomas with “active” necrobiosis can occur as late as 3 years after colon cancer surgery and can be a diagnostic pitfall that significantly affects the management.

## Highlights

• Postsurgical necrobiotic granulomas of the bowel present a diagnostic challenge.
• Granulomas with “active” necrobiosis can occur as late as 3 years after colon cancer surgery.
• Postsurgical necrobiotic granulomas of the bowel can be a serious diagnostic pitfall.

## 1 Introduction

Postsurgical necrobiotic granuloma has been well described in some body sites such as the prostate, bladder, and female genital tract [1,2]. The early lesions mimic rheumatoid nodules and necessitates exclusion of an infectious cause. The lesions are characterized by a central area of brightly eosinophilic fibrinoid necrosis surrounded by palisading histiocytes as well as lymphocytic inflammation and scattered multinucleated giant cells. At a later stage, the lesions heal due to fibrosis [3]. Surgery-related diathermy and traumatic tissue damage are the chief etiologic factors in pathogenesis of the granulomas [1]. Foreign body-type granulomas reactive to suture material have been reported in several body sites [4]. In addition, non-necrotizing granulomatous lesions involving the draining lymph nodes in colon cancer have been well described as a reaction to tumor antigens [5]. However, the occurrence of postsurgical necrobiotic granulomas in the bowel is rare and represents a significant diagnostic challenge. In line with the SCARE criteria, we present the case of a 28-year-old woman with postsurgical necrobiotic granulomas of the bowel, masquerading as ileal deposits, 3 years after colon cancer surgery [6]. The non-classical late presentation of the necrobiotic granulomas and mimicry of the lesions with several entities, both clinically and microscopically, presented a significant diagnostic challenge that prompted us to report this case.

## 2 Report of the case

**Fig. 1:**
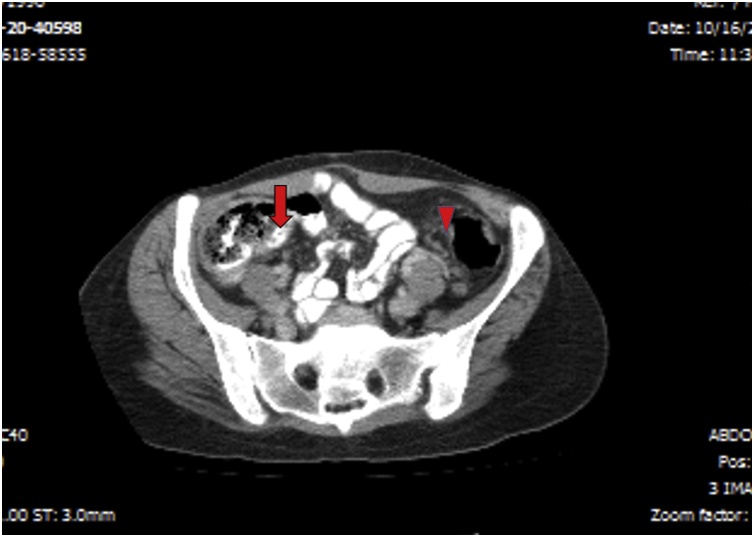
A tumor in left colon (arrow head) and ileal nodules (arrow) in abdominal CT.

**Fig. 2:**
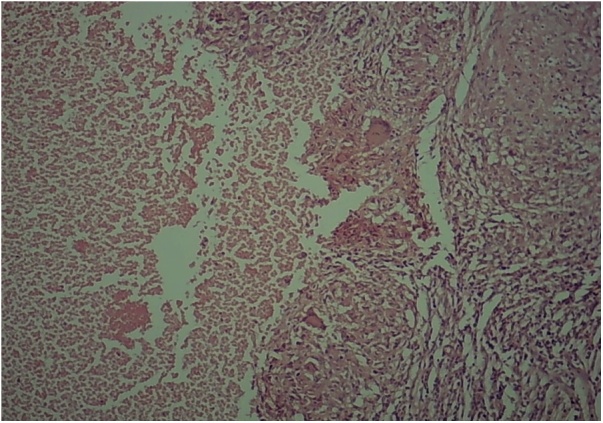
H&E image shows palisading granulomatous reaction surrounding geographic fibrinoid necrosis. Langhans giant cells are seen (100×).

A 28-year-old woman was admitted to our hospital with complaints of constipation, hematochezia, and symptoms of iron deficiency anemia since one month. History revealed that the patient had undergone sigmoidectomy for management of colon cancer before 3 years and was asymptomatic until the last month. On physical examination, the patient was pale and there were no palpable abdominal masses. Biological tests revealed normal results except for low hemoglobin level of 8 signifying anemia. Tumor markers in the blood were within normal limits. CT scan revealed ileal wall nodules and left colon wall thickening (Fig. 1). Endoscopic biopsies were positive for well-differentiated adenocarcinoma in both the right and left colon segments. Total colectomy under general anesthesia was performed, and the exploration was unremarkable, with the exception of suspicious tumor deposits in the ileum. The surgeon performed resection of the involved ileal segment. The surgery was uneventful with no hemorrhage or peritonitis. Upon histopathological examination, the colon specimen was signed out as “Recurrent multifocal moderately differentiated adenocarcinoma, rpT3(m)N1” [7]. The ileum specimen showed only necrotizing granulomas in the form of foci of fibrinoid necrosis surrounded by palisading histiocytes and lymphocytes as well as scattered Langhans giant cells (Fig. 2). Ziehl-Neelsen and PAS special stains were performed, which showed negative results. Extensive search did not reveal microscopic vasculitis. Furthermore, inflammatory bowel disease was excluded histopathologically and clinically. Thorough clinicoradiological workup including the purified protein derivative (PPD) test excluded tuberculosis, sarcoidosis, and autoimmune rheumatic or vascular disease. The patient was given 5 cycles (out of 12) of FOLFOX (Folinic acid-5-Fluorouracil-Oxaliplatin) chemotherapy over the 5 months after operation. Follow up by CT and the tumor markers CA19-9 and CEA at 8 months postoperation showed normal results.

## 3 Discussion

Postoperative necrobiotic granulomatous reactions have been described in the prostate, bladder, female genital tract, and thyroid [[1], [2], [3],^8^,^9^]. These reactions are often accompanied by foreign body-type granulomas [3]. However, in the present case, we did not find foreign body-type giant cell reaction. The lesions were solely composed of geographic areas of brightly eosinophilic necrotic material surrounded by palisading histiocytes and scattered Langhans giant cells. The same bowel segment that involved the granulomatous reaction did not show adenocarcinoma. Moreover, the colon specimen that involved the adenocarcinoma was negative for granulomas. These features led to the initial impression of caseating granulomas. However, special stains and thorough clinicoradiological workup excluded an infectious etiology. Further search in the patient’s history did not reveal any rheumatic or other possible autoimmune diseases. Interestingly, a previous study has reported non-infectious necrotizing granulomas in the absence of a history of autoimmune disease or previous surgery [10]. The plausible explanation for the histomorphological features in the present case was an iatrogenic pathology related to the previous colon surgery. Electrocauterization was suggested as the common etiologic factor in other organs for postoperative necrobiotic granulomas [1]. However, the location of the lesions in the present case makes the aforementioned etiology less applicable and suggests a minor trauma during previous surgery as the possible pathogenetic factor. Another interesting finding in the present case was the occurrence of granulomas accompanied by recurrence of the cancer. Granulomatous reactions associated with colon cancer have previously been documented; however, these were not associated with necrosis [5]. Granulomatous reactions in lymph nodes draining cancer are mainly of the foreign body type and can be attributed to tumor antigens. Geographic fibrinoid necrosis is generally not found in such lesions [11]. We considered tuberculosis, rheumatoid nodules, inflammatory bowel disease, and vasculitis in the histopathological differential diagnosis of the present case. All diseases were excluded based on negative past medical history for autoimmune disease and microbiological studies, and absence of histopathological features indicating inflammatory bowel disease or vasculitis.

## 4 Conclusion

In conclusion, granulomas with “active” necrobiosis can occur as late as 3 years after colon cancer surgery and can be a clinical and microscopic diagnostic pitfall that significantly affects the management. The current case is an unusual presentation in a young woman with colorectal adenocarcinoma. Further molecular and biomarker studies are needed.

## Funding

This research did not receive any specific grant from funding agencies in the public, commercial, or not-for-profit sectors.

## Ethical approval

Not applicable. This case report is exempt from ethical approval in our institution.

## Consent

Written informed consent was obtained from the patient for publication of this case report and accompanying images.

## Registration of research studies

NA.

## Guarantor

Ali Al Khader.

## Provenance and peer review

Not commissioned, externally peer-reviewed.

## CRediT authorship contribution statement

**Ali Al Khader:** Conceptualization, Data curation, Investigation, Methodology, Supervision, Validation, Visualization, Writing - original draft, Writing - review & editing. **Esra Nsour:** Investigation, Methodology, Validation, Writing - original draft, Writing - review & editing. **Raed Aldabbas:** Data curation, Investigation, Methodology, Writing - review & editing. **Anwar Alneweiri:** Data curation, Resources, Writing - review & editing.

## Declaration of Competing Interest

The authors declare that they have no conflict of interest.
